# Wheat Biocomposite Extraction, Structure, Properties and Characterization: A Review

**DOI:** 10.3390/polym13213624

**Published:** 2021-10-21

**Authors:** Abdulrahman A. B. A. Mohammed, Abdoulhdi A. Borhana Omran, Zaimah Hasan, R. A. Ilyas, S. M. Sapuan

**Affiliations:** 1 Department of Mechanical Engineering, College of Engineering, Universiti Tenaga Nasional, Jalan Ikram-Uniten, Kajang 43000, Selangor, Malaysia; rahman.aziz@uniten.edu.my (A.A.B.A.M.); zaimah@uniten.edu.my (Z.H.); 2 Department of Mechanical Engineering, College of Engineering Science & Technology, Sebha University, Sabha 00218, Libya; 3 School of Chemical and Energy Engineering, Faculty of Engineering, Universiti Teknologi Malaysia, Johor Bahru 81310, Johor, Malaysia; 4 Centre for Advanced Composite Materials (CACM), Universiti Teknologi Malaysia, Johor Bahru 81310, Johor, Malaysia; 5 Laboratory of Biocomposite Technology, Institute of Tropical Forestry and Forest Products (INTROP), Universiti Putra Malaysia, Serdang 43400, Selangor, Malaysia; 6 Advanced Engineering Materials and Composites Research Centre (AEMC), Department of Mechanical and Manufacturing Engineering, Universiti Putra Malaysia, Serdang 43400, Selangor, Malaysia

**Keywords:** wheat biocomposite, wheat starch, wheat gluten, wheat fiber, antioxidant, antimicrobial

## Abstract

Biocomposite materials create a huge opportunity for a healthy and safe environment by replacing artificial plastic and materials with natural ingredients in a variety of applications. Furniture, construction materials, insulation, and packaging, as well as medical devices, can all benefit from biocomposite materials. Wheat is one of the world’s most widely cultivated crops. Due to its mechanical and physical properties, wheat starch, gluten, and fiber are vital in the biopolymer industry. Glycerol as a plasticizer considerably increased the elongation and water vapor permeability of wheat films. Wheat fiber developed mechanical and thermal properties as a result of various matrices; wheat gluten is water insoluble, elastic, non-toxic, and biodegradable, making it useful in biocomposite materials. This study looked at the feasibility of using wheat plant components such as wheat, gluten, and fiber in the biocomposite material industry.

## 1. Introduction

Plastic materials cause significant environmental damage and are one of humanity’s greatest issues. Petroleum-based plastics are non-biodegradable, even after a hundred years. Plastic polymers, which are created from non-renewable elements, are one of the primary causes of global warming. Biocomposite materials are the ideal choice for possibly replacing fossil-based polymers. However, biocomposite materials require further development in terms of their characteristics [1].

Improving the properties of biocomposite material is still being investigated by researchers [2,3,4,5,6,7]. There is an abundance of research on wood and non-wood plants to extract starch, gluten and fiber in order to produce bio-composite materials. The ingredients of biocomposite materials are extracted from various types of agricultural crops, such as wheat, corn, cassava, hemp, jute, kenaf and other crops [8]. The advantages that make plants more useful than other sources for biopolymers are their availability, quality and quantity. In addition, plants offer variation in physical properties such as thickness, density, water content, water absorption and water solubility. There exists a variation in chemical constituents such as cellulose, hemicellulose, lignin and protein content in fiber, amylose and amylopectin ratio in starch [9]. Furthermore, their diversity in degree of polymerization, degree of crystallinity, water-vapor permeability and porosity make a difference in the biocomposite properties.

Wheat is a non-wood plants based fiber [10], which is planted in many countries and produces a lot of waste. Starch is the primary component of wheat, having a number of food and industrial applications [11]. In biocomposite application, wheat starch is used as biopolymer film with or without filler. Wheat fiber can be extracted from different parts of the plant to be used as reinforcement filler for either natural or synthesis matrix. Surface treatment is a method that is commonly used to clean, modify and improve the fiber surface to decrease surface tension and to improve the interaction between the fiber filler and the starch film matrix or synthesis matrix [12,13,14,15,16]. Several publications have addressed the effects of sodium hydroxide treatment on the structure and properties of natural fibers such as kenaf, flax, jute, hemp, sugar palm and wheat fiber [17,18,19,20,21,22].

Straws such as wheat, rice and rapeseed straws, which known as cereal straws, are not only highly abundant but they are also a low-cost, potential candidate to be utilized in the development of green composites [23]. Wheat is one of the crops that is most sought after, and it is widely cultivated. The source of it comes from a grass named (*Triticum*) that is grown in countless countries around the entire globe. The total production of wheat in 2019–2020 was 763.9 million metric tons [24] and this percentage increases yearly.

One of the co-products from the starch and bioethanol industry is wheat gluten, which is utilized in many food and non-food application. It is widely used to develop films and other Bioplastics [25,26,27,28,29]. In 36 days, the decomposition of wheat gluten takes place in aerobic fermentation and takes 50 days in farmland soil without releasing any toxic residues into the environment [30]. Wheat gluten protein has a high decomposition rate, even when it is subjected to chemical and physical treatments. Therefore, wheat gluten polymer is a perfect alternative for the development of new biodegradable polymers, because of its decomposition properties and its unique viscoelastic and gas barrier properties [31]. Furthermore, wheat gluten has been explored as a raw material for non-food applications such as biopolymers [32,33,34]. In order to develop the eco-industry on our planet, biodegradable materials such as wheat-based biocomposites, which are distinguished with unique advantages such as, renewability, availability and low-cost raw materials.

Plasticizers used with starch to create the polymeric entangled phase, by reducing intramolecular hydrogen bonding [35,36,37]. Adding plasticizer to wheat starch improves the physical and mechanical properties because plasticizer increases the flexibility of the material. There are many types of plasticizer such as, fructose, sorbitol, urea and glycerol used to improve physical and mechanical properties. Similarly, to enhance mechanical and physical properties, plasticizers have been applied in many biocomposite materials, such as corn [38,39,40], sugar palm (*Arenga pinnata*) starch [41], cassava [42] and rice starch [43,44].

In this work, we conduct a comprehensive study on wheat fiber, as well as wheat starch biopolymers. This review paper will reveal the improvement of the properties in terms of mechanical response, thermal behavior, antioxidant, antimicrobial, and morphological properties of different parts of the wheat plant that can be used as a bioplastic material.

## 2. Wheat Plant

Wheat is a grass plant of the *Poaceae* plants family; the scientific name of wheat plant is *Triticum*. Wheat is one of the world’s most ancient and essential cereal crops, which is grown across a wide range of climates and types of soils [45].

**Figure 1:**
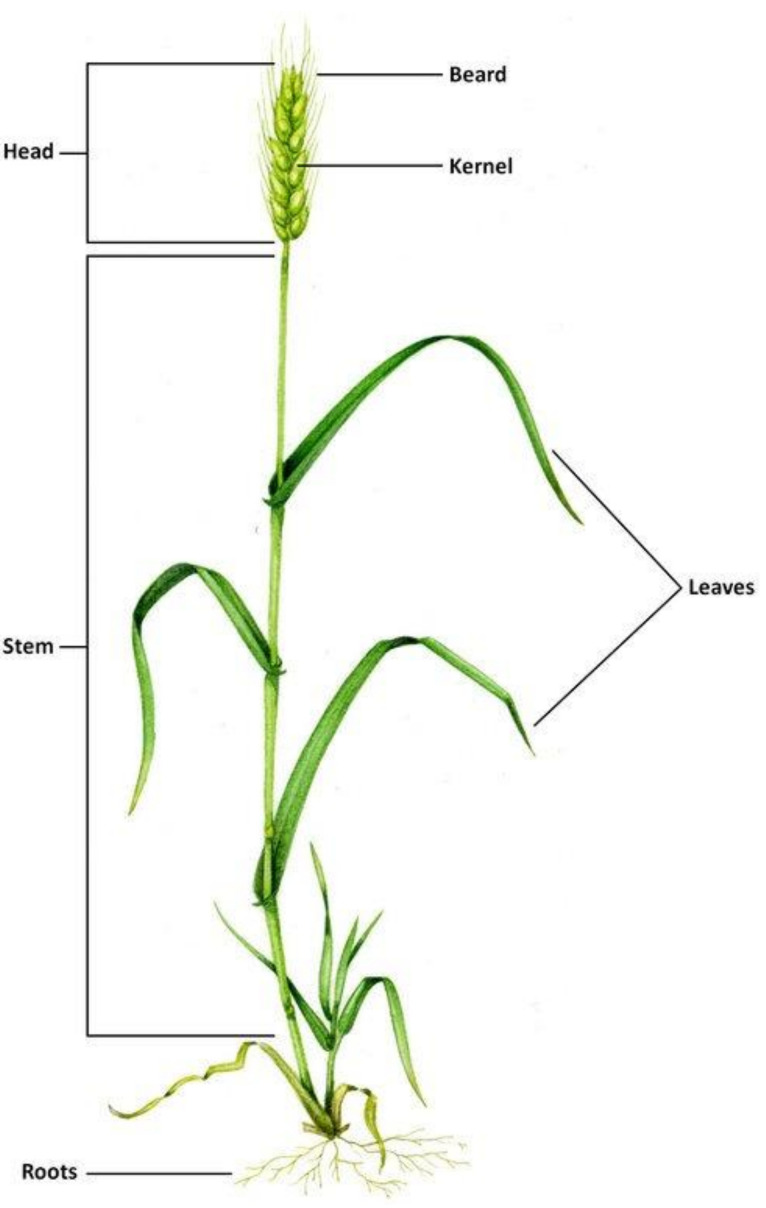
Wheat plant main parts [48].

The main parts of the wheat plant are head spike, stem, leaves and roots. Wheat plants grow up to 2–4 feet tall. Figure 1 shows wheat plants’ main parts. The kernel of the wheat (also called the wheat berry) is the seed of the wheat plant [46], while the part that covers the kernel and protects it is called the beard; similar to all the grass plants, wheat plants stand on the stem. The leaves of wheat plants are long and comparatively thin; flog leaves are in the top of the leaves, which are responsible for the protection of the leaves. The nourishment from the soil to the plant comes through roots in the bottom of the plant [47].

## 3. Film Preparation and Properties Characterization of Films Based Wheat Starch

There are many factors that affect biopolymer properties, including: starch type, treatment temperature, additions such as plasticizer and co-biopolymers [35]. In this section, we will discuss properties of film-based wheat starch.

### 3.1. Physical and Chemical Properties of Wheat Starch

Wheat is one of the most widely farmed crops worldwide; the type of the soil and soil-dryness conditions affects the quality of the starch and other plant parts. The gelatinization enthalpy and swelling power of moderate soil-dryness treated starch are low. When compared to well-watered conditions, however, a greater gelatinization temperature, retrogradation enthalpy, and retrogradation percentage are found. According to Weiyang Zhang et al. [49], soil dryness affects amylose structure more than amylopectin structure in wheat grains. Furthermore, moderate soil dryness improves molecular structure and functional properties of the starch. Table 1 shows a comparison between the chemical and physical structure of wheat, corn, rice and potato starches. There is no significant difference between the chemical composition of various starches.

The starch basically contains Amylose and Amylopectin. In biocomposite materials, it is important to identify the percentage of Amylose and Amylopectin, which directly affect the properties of the film or the matrix of the bio-polymer [50]. Amylose has a lower molecular weight than amylopectin; however, the high relative weight of Amylopectin reduces the mobility of polymer chains, resulting in high viscosity, whereas the linear structure of Amylose has demonstrated behavior more similar to that of conventional synthetic polymers [51]. The majority of natural starches are semicrystalline. Depending on the resource of the starch, the crystallinity of starch is around 20–45% percent. The short-branched chains in Amylopectin are mostly responsible for crystalline regain and appear as double helices with a length of around 5 nm. In the crystalline areas, the Amylopectin segments are all parallel to the big helix’s axis [52]. Since proteins and polysaccharides are the primary components of natural polymers, the structure–property relationships in these materials are determined by their interactions with water and with each other in an aquatic medium [53].

Thianming Zhu et al. [54] applied different techniques to determine the percentage of Amylose in the starch; techniques included Differential Scanning Calorimetry (DSC), High-Performance Size-Exclusion Chromatography (HPSEC), iodine binding, and Megazyme amylose/amylopectin. Michael Ronoubigouwa Ambouroue Avaro [55] developed a method that used Tristimulus CIE Lab Values and developed a specific color board of Starch-iodine complex solution, the conversion of the regression values L*a*b* to Red, Green, Blue (RGB) values and to color hexadecimal codes. This method used a colorimeter device. A spectrophotometer is another device that can be used to detect the percentage of the Amylose by calculating the absorbent light that gets through the mixture of the starch and iodine solution [56,57,58].

**Table 1:** A comparison between the chemical composition and physical properties of wheat, corn, rice and potato starches [59,60,61,62,63,64,65,66,67,68,69,70,71,72].

	Type of Starch			
Parameter	Wheat Starch	Corn Starch	Rice Starch	Potato Starch
Amylose (%)	16.0–31.5	20.0–28	20–28	25–31
Amylopectin (%)	68.5–75	75–83	65–85	76–83
Ash (%)	0.20–0.29	0.32–0.62	0.17–0.19	15.95–16.05
Proteins (%)	0.40–0.46	0.38–7.7	0.33–0.38	4.26–4.82
Density (g/cm^3^)	1.5	1.356–1.4029	1.282	0.763
Moisture content (%)	10.65–13.3	10.45–10.82	3.60	15.98 ± 0.36

### 3.2. Production of Films Based Wheat Starch

In order to produce starch-based films, starch should be isolated from granules [73], then the isolated starch is mixed with distilled water and plasticizer to prepare the slurry. Subsequently, casting and drying processes takes place.

#### 3.2.1. Wheat Starch Isolation

Zuosheng Zhang et al. [74] discussed different methods of starch isolation, including Alkaline Washing (ALW), Ultrasonic Assist Ethanol Soaking (UAES), Hot Water Soaking (HWS) and Ultrasonic Assist Hot Water Soaking (UAWS). A similar crystalline pattern of C-type was found for all the isolated starch samples; starch isolated using the ALW and UAES methods shows a greater degree of crystallinity than the other isolation methods. FT-IR spectra analysis shows similar chemical interactions with different isolation methods. Starch isolated using the UAES method exhibited the highest water solubility. The HWS and ALW methods resulted in greater enthalpy and gelatinization temperatures, while the UAES and UAWS isolation methods resulted in greater peak viscosity.

According to Ali et al. [75], starch can be isolated from the kernel by soaking 1 kg of flour in four liter of distilled water and keeping the mixture at 4 °C for 12 h. Then the slurry mixture is diluted 10 times (volume/volume) with distilled water. Then a 20 g of sodium hydroxide is dissolved in 1000 mL of water to make 0.5 M of NaOH. The solution of NaOH is then added to the diluted slurry. The diluted slurry is then mixed by continuously stirring for one hour. The starch is filtered and centrifuged for 30 min at 10 °C. The sediment gained from the surface is scraped and the lower white portion recovered as starch and, subsequently, dried at 40 °C in a hot air oven.

#### 3.2.2. Wheat Starch Film Preparation

**Figure 2:**
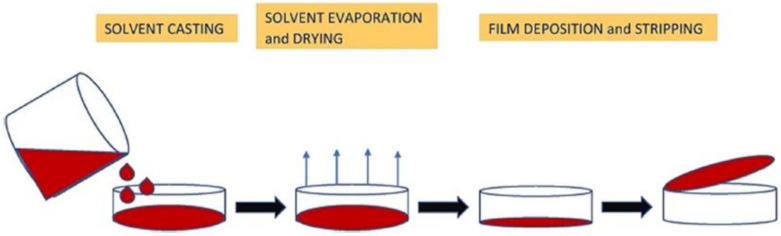
Casting method of film formation [80].

The process of preparing wheat-starch film starts with mixing the pure starch that has been isolated from other kernel ingredients with distilled water. Then the mixture is put on a hot plate mechanical stirrer for full dispersion in a temperature around 80 °C to 100 °C [76,77]. If the process contains the addition of a plasticizer, to ensure high homogeneity in the film, the plasticizer addition is recommended to take place after the starch is dispersed in the distilled water [39]. Once the slurry is cooled to room temperature, the slurry is casted on a petri dish or Teflon mold. However, Teflon mold prevents sticking of the film that happens with petri dishes [78,79]. Subsequently, the slurry is put into a drying oven at 45 °C with air circulation to remove water and moisture [76]. After the film is fully dried, it is peeled off carefully as to avoid rupture. Figure 2 shows the casting method of film formation.

### 3.3. Properties Characteristics of Wheat Starch-Based Film

#### 3.3.1. Pasting Properties

**Figure 3:**
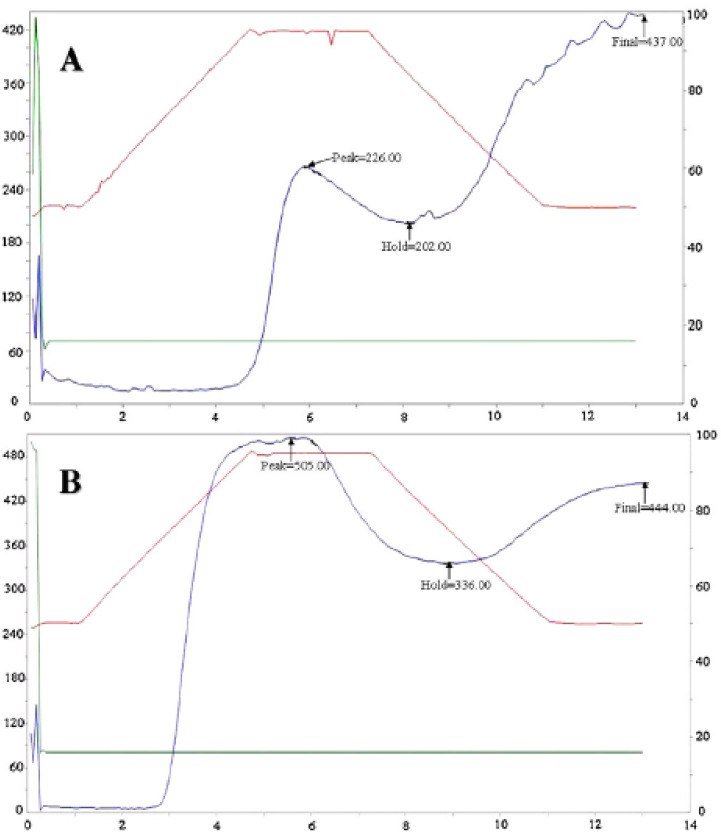
Rapid Visco-Analyzer pasting profiles of (**A**) normal wheat and (**B**) waxy wheat starch [77].

The pasting properties of starch samples can be determined using a Rapid Visco-Analyzer RVA; the properties of pasting viscosity profiles are shown in Figure 3. The process of testing the pasting properties can be undertaken by following H. Liu method, where weighed starch and distilled water is mixed and stirred in the aluminum Rapid Visco-Analyzer RVA sample canister to obtain a 10.0% starch suspension. A programmed cooling and heating is used to record the amylograms of the pastes [81]. Studies by Huanxin Zhang et al. [77] show that the paste viscosity of the waxy wheat-starch was gradually enhanced and reached a peak at 73.6 °C, while the normal wheat starch peak temperature was 94.7 °C.

#### 3.3.2. Morphological Properties

**Figure 4:**
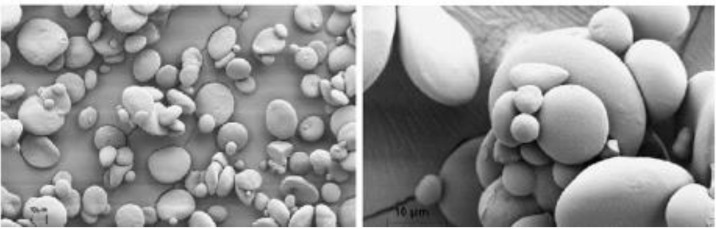
Morphology of native wheat starch [85].

Morphological properties in biocomposite are extremely important in order to see how homogeneous the composite is to get through. The scanning electronic microscope test also gives a structural explanation for other properties. For example, if the film surface is homogenous, this would indicate integrity of other properties. Wheat starch has a bimodal size distribution, with small, round B granules (2–10 mm) and large, lenticular (20–32 mm) [82,83,84]. Figure 4 shows the morphology of the wheat starch.

**Figure 5:**
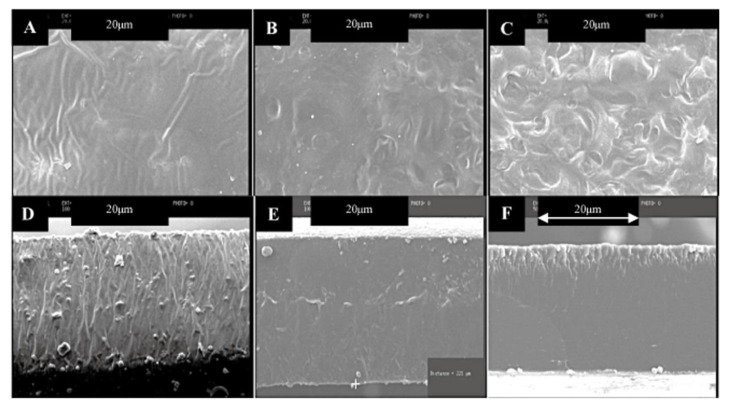
Morphology of biopolymer-based wheat starch (**A**–**C**) represent wheat starch biopolymer surface with 0, 20 and 50% of Glycerol, respectively, while (**D**–**F**) represent wheat starch biopolymer cross-section with 0, 20 and 50% of Glycerol, respectively [76].

Non-plasticized films usually have cracks or pores and some undissolved particles, which could make it easier for water vapor to pass through the film. Plasticizing wheat films with Glycerol reduced those cracks and pores. Plasticizing also improves the adhesion between the particles of the material, as shown in Figure 5. Similar results were reported for plasticized starch-based films such as corn starch-based films [86], cassava starch-based films [42], sago starch-based films [87], rice starch-based films and potato starch-based films [88].

#### 3.3.3. Film Transparency

The film transparency (reverse of opacity) is used to manifest the ability of light to pass through the film. Films that have a low degree of opacity are usually referred to as being acceptable as packaging material because they offer the better visual view of the food [89]. However, the variation of transparency gives more options in different applications. The opacity of the film can be calculated with the equation below:

(1) $$\mathrm{Opacity}=\frac{\mathrm{Abs}600}{x}$$

where: x represents the thickness (in mm) of the film, and Abs600 is the absorbance of light measured at 600 nm [90]. Lower values of the opacity value mean greater transparency.

The bioplastic wheat-starch-based films have lower opacity than that of the corn-starch-based films. This indicates that wheat-starch-based films exhibit higher transparency compared to corn-starch-based films. However, the highest transparency has been found in the potato-starch-based films [88,91]. The high opacity of corn-based film can be attributed to high lipid content of corn-starch film [92], while the addition of protein in starch films also improves the transparency [93]. However, the Amylopectin in potato starch contains a high number of long chains, which contribute to the formation of the compact structure that leads to a more transparent starch matrix [94].

#### 3.3.4. Thermal Properties

Thermal tests are important to gauge the information about the thermal behavior of the biocomposite film. Thermogravimetric Analysis (TGA) is used to measure the temperature change over the time, while Derivative Thermogravimetry (DTG) is used to show the phases degradation of the material [95]. Differential Scanning Calorimetry (DSC) is used to measure the thermal properties of starch such as peak gelatinization temperature (Tp), gelatinization onset temperature (To), gelatinization conclusion temperature (Tc), and enthalpy of gelatinization (ΔH) [96,97]. Wheat starch begins to breakdown at nearly 275 °C, according to research. The temperature at which wheat nanofibers films degraded was roughly 296 °C [98]. Jie Zeng et al. [99] found that the gelatinization onset temperature of wheat starch is 59.43 °C, the peak gelatinization temperature is 64.23 °C, the gelatinization conclusion temperature is 78.02 °C, and the enthalpy of gelatinization is 2.915 J g⁻^1^. Sorghum starch (Broom-corn) thermal properties show a little more peak gelatinization temperatures (Tp) and enthalpy compared to wheat starch, peak gelatinization temperatures (Tp) was reported for sorghum ranging from 68.2 °C to 77.8 °C, while the enthalpy values ranged from 8.2–16.4 J g⁻^1^ [100]. The onset temperature in biocomposites based starch and plasticizer is around 300 °C, the elimination of hydrogen functional groups, degradation, and depolymerization of the starch carbon chains polymer happened at this stage [101], while creating strong bonds by adding additives such as fiber and cross-linkers delays degradation temperature [102].

#### 3.3.5. Water Vapor Permeability (WVP)

Water vapor transmission rate (WVTR) or moisture vapor transmission rate (MVTR) is a measure of the passing of water vapor through the substance. It is a measure of the permeability for vapor barriers. According to ASTM E96-00 standard, the films should be placed in the dryer oven for 48 h under 25 °C and 67% relative humidity before starting the test [103]. WVP is calculated from the transmission of the vapor across the films due to the difference in the partial pressure [104,105,106].

X. Guo et al. [107] tested the ratio of zein to wheat gluten. The researchers found that when the ratio of zein to wheat gluten is increased, the WVP decreases. WVP is related to the protein’s characteristics. Gluten is made up of multiple proteins with more polar residues in the gluten molecules. Zein, on the other hand, contains a higher proportion of hydrophobic residues. For this reason, when the ratio of zein to wheat gluten is increased, WVP decreases.

Plasticizers such as glycerol have a great effect on water vapor permeability. Wheat-starch films without plasticizer have higher WVP compared to plasticized wheat films with 20% and 30% glycerol. However, the WVP of the starch film with 50% glycerol was greater, which can be attributed to micro cracks in the film [76]. The addition of hydroxypropylation, cross-linkers and antioxidants to starch bio-polymers such as corn, rice and wheat starch improves water barrier resistance [108], because the addition of those additives reduce polymer polarity, which results in low hydrogen bonding [109]. Film thickness does not affect the WVP, since the amount of casted solution does not molecularly rearranged during the drying process [110].

#### 3.3.6. Crystallinity

Crystalline substance, in most conditions, exhibits a polycrystalline structure. Each grain being separated from the next one by a boundary, along which the atomic configurations are heavily distorted [111]. X-ray diffraction (XRD) is a technique that is used to analyze and measure the crystalline phases of a different types of substances, basically for mineralogical analysis and identification of unknown substances. Powder diffraction data are fundamentally derived X-ray Diffraction by the atomic and molecular arrangements explained by the physics of crystallography. One advantage of using X-ray diffraction is its ability to characterize crystal index with high-accuracy [112].

Granular starches are partially crystalline because they contains of approximately 25% w/w of the linear polysaccharide amylose and 75% w/w of the branched polysaccharide amylopectin [113]. Starches from various sources have close crystalline index. Corn starch, rice starch, and potato starch have crystalline indexes of 43–48%, 38% and 23–53%, respectively, while wheat starch crystalline index is 36–39% [114]. The relative crystallinity of wheat starch decreased with heat moisture treatment, because the heat moisture treatment disrupt helical structures in the amorphous and crystalline region [115]. Amylose has 33.3% crystallinity index while Amylopectin has 0% crystallinity index, preparing films by blending Amylose and Amylopectin shows co-crystallization between them. Starch-based film shows higher crystallinity than expected, which refers to crystallization of Amylopectin [116].

## 4. Wheat Gluten-Based Film; Preparation and Characterization 

Wheat Gluten (WG) is the primary protein in wheat grains [117]. Films that are made from wheat gluten have potential to develop an edible film, adhesives, binders, and biomedical substances. The main advantages of wheat gluten films include being insoluble in water, elastic in nature, and non-toxic. Gluten matrix is biodegradable and glassy, with characteristics similar to epoxy resin [118,119,120].

### 4.1. Production of Wheat Gluten-Based Film

Wheat-gluten based films can be produced via two common methods:

#### 4.1.1. Wet Method

Wet-type mechanical milling is a common approach for producing nanoparticles for a variety of bio-materials, including starch and gluten [121]. For gluten, a milling process is used to obtain gluten powder. The wheat gluten suspension solution is made by mixing the gluten powder with ethanol (70% aqueous ethanol). Then fibers are immersed in gluten suspension-solution. After the mixture is homogenized, the composite is dried in a vacuum air oven to allow the solvents (water and ethanol) to evaporate more quickly [97].

#### 4.1.2. Dry Method

This method can be performed by either; (1) spreading dry powder with dry fibers in the mold, where the gluten powder will be first distributed in the mold. Next, the dry fiber preforms will be placed into the mold. Subsequently, another gluten powder layer would be added through a sieve. These steps will be repeated until the desired thickness is achieved (2), by spreading dry powder on wet fiber in the mold. In this method, fiber must be wetted again (after combing and drying), as the water will be a processing aid, after casting the gluten powder and wet fiber on the mold, the drying process needs to be conducted in dryer oven [122].

### 4.2. Properties Characterization of Films Based Wheat Gluten

Due to the fact that polar amino acids such as glutamic acid, aspartic acid, lysine, arginine, serine, threonine, and tyrosine are present in proteins, the addition of protein in biocomposite films improves the mechanical properties. Amino acids contain reactive groups that can be useful in cross-linking and creating covalent connections, improving the mechanical characteristics of biocomposites [123]. It has been found that proteins rich in sulfur amino acids, particularly rapeseed proteins when combined with rubber, cause a substantial enhancement of the cross-linking process. Protein-rich composites have a higher thermal resistance due to the high number of nitrogen atoms in a single polypeptide molecule [124].

Wheat-gluten films revealed lower water absorption (settled on 80% after 4000 min), this amount of water absorption is a response for (C=O, C=C) bonds existence in gluten film [125]. While the starch-based films revealed higher water absorption, which reached approximately 520% after 210 min on cassava-starch-based films [42] and 295% after 240 min on corn-starch-based films [126]. All starch-based films showed very strong water absorption capacity. However, the amount of absorbed water is different from one starch to another. This behavior is attributed to the size of starch particles, the smaller the particles the earlier and higher water absorption. Also FTIR analysis shows hydrogen bonded hydroxyl group peak more intensely with small-particle content compared to the larger particles, this explains the increase in water absorption capacity [127]. Wheat-gluten-based films, plasticized with glycerol show elongation at break in the range from 320.5–474.5%, 6.33 MPa tensile strength, while the moisture content was just about 5% [128]; the addition of a plasticizer reduces hydrogen bonding, which allows molecules to move and increase the elongation, while the high tensile appears when starch-starch hydrogen bonds overcomes starch-plasticizer bonds in a low amount of plasticizer [129]. Reinforcing wheat-gluten with flax fiber improves the tensile strength and the elastic modulus, because of the hydrogen bonding between the fiber and the protein [122,130,131,132,133]. Heat treatment of wheat gluten at temperature higher than 100 °C reduces the effect of the reinforcing filler which reflected as reduction in the Young’s modulus. This explains the reduction of wheat gluten adhesion when it is heat treated [134]. However, treating the filler with alkaline and/or silane improves adhesion between wheat gluten and filler. This surface treatment increases the mechanical properties by reducing the fiber pullout length [135] As confirmed by FTIR results, fiber chemical treatment removes lignin and hemicellulose and reduces the hydrophilic nature of the fiber and, hence, improves the interfacial adhesion between fiber and matrix [136,137]. Natural structures of bio-polymers have relatively low degradation temperatures [138]. This refers to the low energy level required to break the weak interactions between the polymer chains. To avoid undesirable decomposition of wheat-gluten-based bioplastics, hydrophobic liquids, e.g., castor or silicone oil are used [139,140]. Blending gluten with hydrophobic polymers, such as polyvinylalcohol improves the degradation temperature [25,141]. The addition of hydrophobic polymers widens the gap between the energy required to break bond interactions and the energy required to cause chains breakdown. Although wheat-gluten-based films also prepared with solution cast method, compression molding have given better properties [142]. The wheat-gluten films reinforced with fiber filler can be prepared either by wet or dry method:

Tensile strength increased when drying temperature increased at 35% RH, while it decreased when temperature increased at 70% RH [143]. N. Vo Hong et al. [120] used water as a processing aid together with the use of unidirectional flax fibers to obtain the strongest properties in the fiber direction. Pakanita Muensri et al. [144] found that lignin content in the fibers does not affect the fiber/matrix adhesion. The type of wheat proteins and compression molding conditions controls the properties of wheat-protein films [145]. To make edible films out of wheat gluten, Francisco Zubeldía et al. [146] employed the dry process. They observed that molding temperature has a greater impact on the films’ ultimate mechanical and physical properties than mixing time. This was due to increased disulfide bonding during heating, resulting in a more cross-linked polymeric network, according to the study. Further work needs to be undertaken to understand the mechanism of cross-linking wheat gluten with fillers [147].

## 5. Wheat Fiber

Wheat fiber is an isolated dietary fiber made from the wheat plant. This fiber goes through a special thermo-physical process followed by milling, sieving, and standardizing into application specific grades. Wheat fiber is a white to light beige, fibrous, and odorless powder [148]. Wheat plant is a good source of fiber from different parts, most fibers extracted from wheat husk, straw, and barn. Table 2 and Table 3 show comparison of wheat, corn and rice fibers from husk and straw based on their properties, while Table 4 shows wheat bran properties. Wheat straw and husk show high amounts of cellulose, therefore, they consider as a good source for nano and microfibers.

Jing Huang et al. [149] illustrated the relationship between the data from the chemical method and Near-Infrared (NIR) to identify the fiber chemical composition. The analytical methods that are used to analyse the NIR results were the partial least squares (PLS) and principal component regression (PCR). PLS is proved to be a better quantitative method than PCR [150]. The fiber composition can also be identified through Neutral Detergent Fiber (NDF), Acid Detergent Fiber (ADF), and Acid Detergent Lignin (ADL) [151,152].

Wheat straw has a high amount of cellulose and offers several advantages over the other types of reinforcement fillers. The advantages include being low in density, nonabrasive nature, low cost and having accessibility and renewability [153]. Wheat straw fibers were utilized by Beatriz Montano-Leyva et al. [119] to modify the mechanical characteristics of wheat gluten-based film. By adopting a solvent-free method, the ultimate cost of the materials was lowered. Increases in fiber content of up to 11.1% result in increases in Young’s modulus and stress at break, as well as a reduction in strain at break [154].

Yi Zou et al. [155] used long and untreated wheat straw fiber (WS) (10 cm) with polypropylene (PP) webs to develop a lightweight and cost-effective thermos-plastic composite. In this study, whole straw and split straw have been compared. Split WS–PP composites have improved over whole straw composite by 39% in modulus of elasticity, 69% enhancement in tensile strength and 18% improvement in impact resistance properties, 26% enhancement Young’s modulus, 69% improvement in flexural strength. Comparing lightweight WS–PP composites with Jute–PP composites of the same density, showed that, mechanically split WS–PP composites have 114% improvement in flexural strength, 38% improvement in modulus of elasticity, 140% improvement in Young’s modulus, 10% enhancement in tensile strength, better sound absorption properties and 50% lower impact resistance.

Other applications of wheat straw include extracting off hemicellulose from wheat straw (WS) and it is used as reinforcing filler for kappa carrageenan-locust bean gum polymeric blend films [156]. Wheat straw is also used as reinforcement fiber and injected with polylactic acid (PLA), PLA–WS (70:30) [157]. Additionally, wheat straw is also used with thermoplastic resins to improve their properties [158,159] and used in thermosetting resins-straw boards [160,161].

Wheat bran is the hard outer layer of cereal grains [162]. Lucia Fama et al. [163] reinforced cassava matrix with wheat bran; they found that the interaction between starch and fillers increased with the availability of hydroxyl groups in the film, which involved in a dynamic exchange with water. Zong-qiang Fu et al. [164] used wheat bran as a filler with corn starch matrix. WVP is poor in starch-based films that are not supplemented with wheat bran fiber. By increasing the wheat bran fiber content, the elongation at break of films is decreased. The tensile strength increased up to 10% w/w (up to 5.07 MPa) with the addition of wheat bran fiber content, then declined when the wheat bran fiber content was increased.

Lucia Fama et al. [163] reinforced cassava matrix with wheat bran, they found that the interaction between starch and fillers increased with the availability of hydroxyl groups in the film, which involved in a dynamic exchange with water.

Due to the strong mechanical properties and biocompatibility of isolated cellulose, it is gaining a lot of interest as a reinforcing material [165]. However, in comparison to all-cellulose composites (ACCs), where the reinforcement and matrix are both cellulose, reinforcing polymers with cellulose gives relative poor dispersion of cellulose with synthesis and bio-matrix resulted in reduced interfacial affinity [166].

**Table 2:** Chemical structure and physical properties of wheat, corn and rice husk [167,168,169,170,171,172,173,174].

	Type of Husk		
Parameter	Wheat Husk	Corn Husk	Rice Husk
Density (g/cm^3^)	0.75	1.49–1.18	0.1214
Moisture content (%)	6–6.05	7.6–8.7	9
Cellulose (%)	36–39.2	31.3–47	34.34–43.80
Hemicellulose (%)	18–26.4	34–43.91	19–25
Protein (%)	6	7	1.70–7.26
Fats (%)	5	17.2	0.38–2.98
Lignin (%)	6.8–16	1.5–14.3	16

**Table 3:** Chemical composition and physical properties of wheat straw [175,176,177,178,179,180,181,182,183].

	Type of Straw		
Parameter	Wheat Straw	Corn Straw	Rice Straw
Density (g/cm^3^)	0.3231–0.871	0.033–0.069	0.194
Moisture content (%)	8–60	25–30	6.58–18
Cellulose (%)	28.8–51.5	28–44	29.2–38
Hemicellulose (%)	10.5–39.1	36.05–36.83	12.0–29.3
Protein (%)	3–6.3	4–9	3–7
Lignin (%)	5.4–30	7–29	12–19.0

**Table 4:** Chemical composition and physical properties of wheat bran [184,185,186,187,188].

Wheat Bran	
Parameter	Amount
Density (g/cm^3^)	0.17–0.25
Water holding capacity (g/g)	3.39–6.49
Water retention capacity (g/g)	2.17–5.76
Moisture content (%)	8.2
Cellulose (%)	11.65–13.15
Hemicellulose (%)	49.7
Starch (%)	55.9–70.53
Protein (%)	15.8–16.88
Lipid (%)	3.8–4.13
Lignin (%)	5.3

## 6. Antioxidant Properties of Wheat Based Film

The inhibition of oxidation improves the stability of polymers to be effective in more applications [189]. The addition of antioxidant into films can change the structure of the film [190], where the reduction in the antioxidant impairs the resistance to degradation [191]. Antioxidant materials are added to prolong the useful life of the constituents of polymers [117,192], the polymer type and the compound formulation and the end use application are governing the selection of the correct combination of antioxidants [193]. Wheat starch–chitosan films show the highest antioxidant (α-tocopherol) capacity. However, the addition of α-tocopherol led to more heterogeneous film structure [194]. Feruloylated arabinoxylans extracted from wheat bran show high antioxidant activity in the presence of bound ferulic acid [195].

## 7. Antimicrobial Properties of Wheat Based Film

Antimicrobial property has received more attention recently, especially in the bio-packaging food industry [196]. It has been found that composite wheat gluten-chitosan-based films can prevent microbial growth in intermediate-moisture conditions [197], where gluten is thought to act as an antimicrobial agents carrier [198,199,200]. Organic acids, enzymes, various plant extracts, bacteriocins, and essential oils have been integrated into biopolymers as antimicrobial agents [201,202,203]. Essential oils (EOs) used in food packaging films to inhibit the growth of bacteria and fungi [204,205,206]. Essential oils are natural, volatile, complex compounds with a strong odor extracted from plants [207]. They have health benefits, antimicrobial and antioxidant properties [208,209]. (EOs) used to reinforce bio-matrix composites [210], such as reinforcing corn wheat starch matrix with lemon oil, and the addition of lemon oil, significantly increased antimicrobial activity [211]. However, the addition of (EOs) concentration reduced the tensile strength, while the elongation at break does not change [212]. Potassium Sorbate (PS) has been used as an antimicrobial agent for wheat gluten films. (PS) shows antimicrobial activity, but it has been found that when the film is exposed to an absorbing medium, most of the PS is released [213]. Thymol has been added as an antimicrobial to hydroxyethyl cellulose wheat-starch-based films and the results show the film kept the same chemical properties, whereas mechanical properties improved [214].

## 8. Wheat Biocomposite

### 8.1. Wheat Biocomposite Advantages and Applications

One of the significant advantages of agriculture-based biocomposites’ resources such as wheat, is the renewability of agriculture crops; this advantage is limited in forest-based biocomposite plants, unless the green cover of forest is constantly replaced and renewed.

In many countries around the world, wheat is considered the main ingredient of their diet. In the recent year, wheat consumption has increased at a faster rate than all other cereals, which generates enormous amounts of waste [204]. The waste is increasing with the wheat consumption and production [12]. Fibrous tissue in wheat straw reach 67%, which can be considered as a high percentage among cereal plants [205]. Furthermore, wheat has the highest amount of proteins amongst other cereals.

Additional to its application in bioplastics, wheat gluten can be used as a binder with fibers [206]. As the mechanical and physical properties of starch and wheat-gluten-based biocomposites improved with fiber reinforcement [207], these improvements in the properties with the reduction of moisture content due to the addition of wheat gluten make the wheat based biocomposites a good choice in various applications, such as food packaging and drug delivery systems [208,209,210]. Furthermore, starch-based biocomposites foam is used to produce ecofriendly food containers and bioplastic sheets [37]. The abundance of wheat fiber make it a good choice to be included in various applications including printed circuit boards (PCB), cars, interior components, mobile phone casing and other various fields. Besides, wheat straw has been used as a filler in biodegradable matrices to make different products such as diches and trays; it can also be used with other different types of matrices such as thermosetting matrices, and thermoplastic matrices. Wheat biocomposites are found useful for indoor building insulation applications [211,212,213] as they are proven to be environmentally friendly and contribute to cost and energy savings [144,214,215]. Producing micro- and nano-composites separating from wheat wastes, would be one of the conceivable advancement in biocomposites-based wheat such as reinforcing thermoplastic starch polymer with wheat straw nanofibers [85], while the effect of agronomical aspects in micro- and nano-biocomposites needs more investigation [216].

### 8.2. Wheat Biocomposite Fabrication

The fabrication of biocomposite materials by reinforcing natural lignocellulosic fibers (e.g., sugar palm, water hyacinth, sisal, ginger, cotton, sugarcane bagasse, flax, jute, hemp, arrowroot, banana etc.) with polymer composite is frequently advocated to enhance agricultural materials [215,216,217,218,219,220,221,222,223,224,225]. Natural fibers have key advantages such low price, fully biodegradability, high tensile strength and stiffness, and non-abrasive behavior during processing and high availability with worldwide existence of sources [6,226] According to Azammi et al. [227], the mechanical properties of fiber reinforced polymer composite are depend on 4 factors such (1) fiber type, (2) content/loading of fiber, (3) the orientation and dispersion of fibers within the polymer matrix, and (4) the adhesion at the interface between the polymer matrix and fibers. Suitable type of fiber and optimum fiber loading, as well as good orientation and dispersion of fiber within polymer would result in good adhesion, in which ensures a good stress transfer from the matrix to the filler.

Ecological concerns in recent years have been directed at encouraging the development non-food sources for a new materials from renewable sources. Wheat gluten was effectively employed as a by-product of the starch industry for the manufacturing of environmentally friendly agricultural materials. This is due to its biodegradability [228], non-ecotoxicity [228], high availability at a reasonable price (1.4–1.8 USD/kg), as well as intriguing practical features including adhesion characteristics and efficient lipid barrier properties [229], gases [230], and aroma compounds [231]. Besides that, due to its excellent film and thermoplastic qualities, wheat gluten-based products can be produced through either compression molding [232,233], extrusion [234,235] or casting [98,236]. However, due to the high glass transition temperature of wheat gluts, the inclusion of hydrophilic plasticizers is frequently essential for thermal processing and film flexibility. The inclusion of plasticizer within the wheat gluten would resulting in changes of mechanical properties. Various researchers [237,238,239] found that the inclusion of plasticizer within the wheat would improve the elongation at break and reduce the strength at break and Young’s modulus. Therefore, in order to improve the mechanical, water-barrier, thermal and physical properties of plasticized wheat-gluten-based materials, natural and synthetic fibers were introduced. This is undertaken in order to find new balances between process needs and material stiffness preferences [222,240,241] The addition of protein in biocomposite as a component improves their mechanical properties [124].

**Figure 6:**
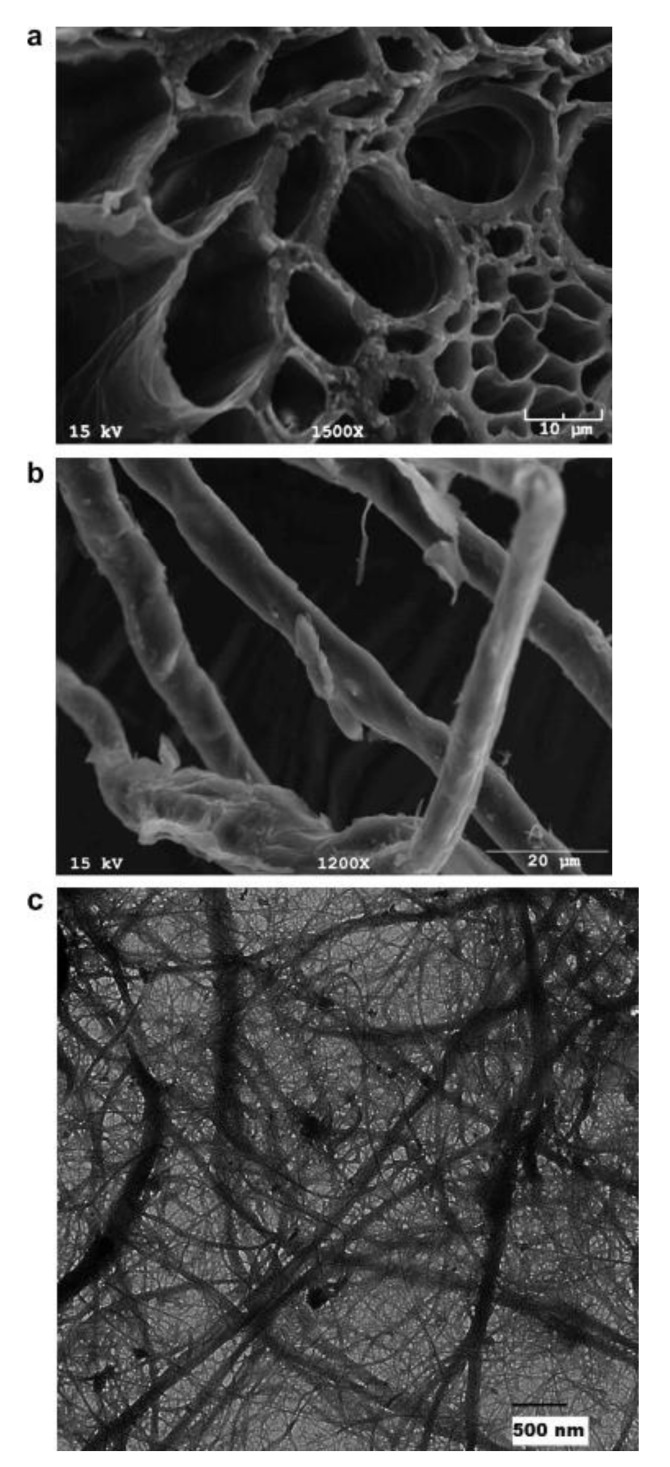
SEM images of the wheat straw cross-section (**a**), microfibers (**b**), and TEM images (magnification ×15,000) of the wheat straw nanofibers (**c**) [98].

Table 5 displays the fabrication, filler loading and optimum mechanical properties of wheat biocomposite. From Table 5, it can be observed that many studies have been conducted on wheat biocomposite. Various techniques have been utilized to fabricate wheat composite such as solution-casting, mixing and compression molding, extrusion and compression molding, and extrusion and injection molding. Usually, the selection of method is based on the final product of the composite, such as film or mold composites. Besides, various polymers had been used to be reinforced with wheat fiber such as Modified potato starch, natural rubber, Polyethylene, Ecovio, PHBV, PLA, Polyester resin, and Polypropylene. Reinforcement of wheat with polylactic acid (PLA) shows the highest tensile modulus and tensile strength, with value of 3450 and 61.2 MPa, respectively. Moreover, many researchers also used wheat biopolymer to be reinforced with various filler such as coconut coir, eucalyptus, wheat straw fibers, hydroxyethyl cellulose, chemlali olive pomace, CNCs rice, CNCs oat, and CNCs. Monta et al. [154] conducted study on wheat straw fiber reinforced with wheat gluten. According to Monta et al. [154], this is the first experiment that had been conducted focusing on incorporating processed wheat fiber into wheat gluten. The wheat straw fibers were prepared using impact milling (IM), cut milling (CM) and ball milling (BM) processes. The result shows that the incorporation of 11.1% of IM or CM wheat straw, or 1.2% of BM wheat straw fiber, increased the mechanical performance of the biocomposite. Additionally, wheat nanocellulose reinforced polymer nanocomposite had also been studied by Alemdar [98]. Alemdar [98] conducted a study on the effect of wheat straw nanofibers reinforced with modified potato starch on morphology, thermal and mechanical properties of bio-nanocomposites. The morphological image of wheat straw can be observed in Figure 6a. The diameter of the wheat fiber decreases as it underwent chemical treatment, as displayed in Figure 6b. Subsequently, the isolation process using chemical treatment resulted in the nano-sized diameters of the nanofibers, which are within the range of 10–80 nm with lengths of a few thousand nanometers, as shown in Figure 6c. The wheat straw nanofibers are well distributed in the modified potato-starch biomatrix, according to scanning electron microscopy (SEM) tests. Furthermore, tensile testing revealed that nanocomposites exhibited a 145% increase in tensile strength and modulus compared to pure thermoplastic modified potato starch.

In the second section of Table 5, examples of corn biocomposites have been added to compare it with wheat biocomposites. Reinforcing Polylactic acid (PLA) with corn cob exhibited lower mechanical properties compared with reinforcing PLA with wheat straw, while corn husk shows higher mechanical properties with natural matrix compared with wheat fiber.

Moazzen, N. et al. [242] developed biocomposite by Blending PVA with starch plasticized with glycerol and reinforced with carboxy methyl cellulous (CMC); the results reveal OC=O stretching and C=O stretching functional groups, PLA, CMC and Glycerol have improved the tensile strength and the hydrogen-bonded hydroxyl group of starch has reduced it. The increase of C-O and C-H stretching vibration intensity confirms the addition of cellulose concentration. Thus, the increase in the intensity of these groups indicates the increase in the crystallinity and mechanical properties of the biocomposite [243].

For hybrid wheat composites, Reddy et al. [244] had conducted experimentation on the preparation and characterization of wheat straw/clay reinforced polypropylene hybrid biocomposite. The hybrid biocomposite samples were fabricated through a melt-blending method using a co-rotating twin-screw extruder, and injection molding. The result shows that the increase in wheat straw loading would reduce the resistance for water absorption and increased the flexural modulus. Additionally, the hybridization of wheat straw (30 wt%) and organo-clay (5%) resulted in the increase in flexural modulus of hybrid composite.

**Table 5:** Fabrication, filler loading and optimum mechanical properties of wheat biocomposite.

Polymer	Filler	Fabrication Process	Filler Loading (%)	Optimum Tensile Modulus (MPa)	Optimum Yield Strength (MPa)	Ref.
Modified potato starch	Wheat straw nanofiber	Solution casting	2–10	271 ± 27.4	7.71 ± 0.67	[98]
Wheat gluten	Coconut coir	Mixing and compression molding	10	2.29 ± 0.47	123.2 ± 34.7	[144]
Natural rubber	Wheat bran	Mixing and compression molding	10–50 phr	-	22	[245]
Wheat gluten	Wheat straw fibers	Mixing and compression molding	0–11.1	18.4 ± 2.3	41.7 ± 3.4	[154]
Polyethylene	Wheat Bran	Extrusion	10–50	371	11.5	[246]
Wheat gluten	Hydroxyethyl cellulose	Mixing and compression molding	0–35	70	2.4	[247]
Ecovio	Wheat husk	Mixing and compression molding	13.5	Flexural: 60	Flexural: 0.75	[248]
Wheat gluten	Chemlal olive pomace	Mixing and compression molding	0–20	40	3.5	[249]
Native Wheat	CNCs rice	Solution casting	0.18 g	34.86 ± 3.3	3.64 ± 0.18	[236]
	CNCs oat			56.58 ± 9.06	5.07 ± 0.33	
	CNCs eucalyptus			70.81 ± 8.22	4.32 ± 0.13	
Phosphorylated Wheat	CNCs rice			31.94 ± 1.38	3.78 ± 0.08	
	CNCs oat			24.37 ± 1.5	3.52 ± 0.14	
	CNCs eucalyptus			30.12 ± 0.35	3.08 ± 0.02	
PHBV	Wheat straw fibers	Extrusion and compression molding	20	3100 ± 200	21 ± 2	[235]
PLA	Wheat straw fibers	Extrusion and injection molding	0–40	3450	61.2	[234]
Polyester resin	Wheat straw strands	Mixing and compression molding	25	Flexural: 2427.2	Flexural: 28.21	[250]
Polypropylene	Wheat straw/Clay	Extrusion and injection molding	Wheat: 0–50Clay: 0–5	Flexural: 2400	-	[245]
Fabrication, filler loading and optimum mechanical properties of corn biocomposite						
PLA	Corn Cob	Mixing and compression molding	0–40	3.7	53	[251]
Corn starch	Corn husk	Solution casting	0–8	620	13	[40]
Polypropylene (PP)	Corn stalk	Mixing and injection molding	40	4.3	34.1	[252]

CNCs = cellulose nanocrystals; MPa = MegaPascal; PHBV = Poly(3-hydroxybutyrate-co-3-hydroxyvalerate); PLA = polylactic acid.

## 9. Conclusions

Conventional plastic-based petroleum materials cause significant environmental damage and are one of humanity’s greatest issues. Using wheat starch and wheat residues to produce biocomposite materials is a promising alternative for plastic-based petroleum. Wheat-starch biopolymer and fiber needs more concern and study, due to their availability, highly abundant, renewability, low cost, good properties and the possibility of using many parts of the wheat plant, which makes wheat plants a good resource for different kinds of biocomposite. Therefore, biocomposite-based wheat represents a good opportunity for biocomposites production in the future. Wheat bran and wheat straw are good sources of fiber to reinforce synthetic polymers and biopolymers. Films that are made from wheat gluten provide the potential to develop an edible film, adhesives, binders, and biomedical substances. The main advantages of wheat-gluten films include that they are insoluble in water, elastic in nature, and non-toxic. The production of wheat-starch-based films can be made via two common methods: (1) wet method and (2) dry method, whereas wheat biocomposite can be fabricated using several techniques such as solution casting, mixing and compression, and extrusion and compression. Plasticizer such as glycerol improves the flexibility and physical properties of wheat-starch-based films. Furthermore, the study of the mechanical properties of wheat biocomposite revealed that biocomposites exhibited increase in tensile strength and modulus when incorporated with fiber. Additionally, the influence of different types of plasticizers, chemical treatment, and addition of cross-linking agents for wheat-starch-based composite is not thoroughly evaluated in the literature. Hence, wheat-gluten-based biocomposites need more research work to better understand functionality and mechanical response of wheat-gluten-based biocomposite films. Molecular weight distribution and the mechanism of cross-linking between the proteins and any other additions such as plasticizers and fibers need further investigation.

## Data Availability

No data were used to support this study.
